# Approximating the nuclear binding energy using analytic continued fractions

**DOI:** 10.1038/s41598-024-61389-5

**Published:** 2024-05-21

**Authors:** Pablo Moscato, Rafael Grebogi

**Affiliations:** The University of Newcastle, School of Information and Physical Sciences, Callaghan, NSW 2308 Australia

**Keywords:** Nuclear physics, Applied mathematics, Computational science

## Abstract

Understanding nuclear behaviour is fundamental in nuclear physics. This paper introduces a data-driven approach, Continued Fraction Regression (cf-r), to analyze nuclear binding energy (*B*(*A*, *Z*)). Using a tailored loss function and analytic continued fractions, our method accurately approximates stable and experimentally confirmed unstable nuclides. We identify the best model for nuclides with $A\ge 200$, achieving precise predictions with residuals smaller than 0.15 MeV. Our model’s extrapolation capabilities are demonstrated as it converges with upper and lower bounds at the nuclear mass limit, reinforcing its accuracy and robustness. The results offer valuable insights into the current limitations of state-of-the-art data-driven approaches in approximating the nuclear binding energy. This work provides an illustration on the use of analytical continued fraction regression for a wide range of other possible applications.

## Introduction

At present, although nearly 100 years have passed since the introduction of the semi-empirical ‘Liquid Drop’-based approximation^1–3^, there is still no wide consensus on a physical model that can be used to analytically derive the nuclear binding energy (NBE) *B*(*A*, *Z*) as a function of the atomic mass number *A* and the number of protons *Z*^4^. In particular, the Liquid Drop Model does not provide good approximations for low *A* nuclides as we will show later in this work.

In our recent work^5^, we introduced a novel symbolic regression technique based on continued fractions. Symbolic regression is a unique type of multivariate regression analysis aiming to find a mathematical expression to approximate an unknown target function that would fit a dataset^6,7^. We use this novel technique to establish both lower and upper analytical bounds for *B*(*A*, *Z*). This approach involves the use of an asymmetric loss function and the representation of the unknown function as an analytic continued fraction.

For the upper bound model (UB), we define the loss function $\ell _{UB}$ as follows:

1 $$\begin{aligned} \ell _{UB}= {\left\{ \begin{array}{ll} |err|, &{} \text {if } relErr \le -relErrTol_{UB}\\ (|err|+1)^{4}, &{} \text {otherwise}, \end{array}\right. } \end{aligned}$$

Here, $|err|=|y_{meas}-y_{pred}|$ represents the absolute value of the residual error between the observed value ($y_{meas}$) and the prediction ($y_{pred}$), while *relErr* is the relative error given by $relErr=err \cdot y_{meas}^{-1}$. The parameters $relErrTol_{UB}$ and $relErrTol_{LB}$ denote the acceptable relative error tolerance values for the upper and lower boundaries, respectively (in our work, they were set to $1\times 10^{-4}$).

Analogously, for the lower boundary (LB), we use a similar structure for the loss function $\ell _{LB}$, but with the condition $relErr \ge relErrTol_{LB}$. This approach allows us to establish both upper and lower analytical bounds for *B*(*A*, *Z*).

**Figure 1:**
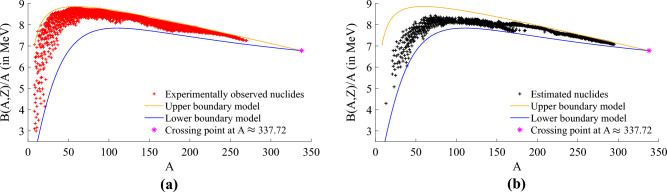
This figure shows that the experimentally observed values from the AME2020^8^ dataset (red cross) are the ones that influence the upper bound model (in orange line) of the nuclear binding energy per nucleon. The lower bound model (blue line) is mainly influenced by the estimated values from AME2020^8^, particularly for low values of *A*. The bounds meet at $A\approx 337.72$. We note that in^5^ we have not made any use of the labels ‘estimated’ and ‘experimental’ so the bounds have been found using the data as a whole. (**a**) Experimentally observed values. (**b**) Estimated values.

In our study^5^, we demonstrated that the models we developed can effectively approximate the bounds of both experimentally observed stable and unstable nuclides, as illustrated in Fig. 1a. This includes nuclides with estimated values, as shown in Fig. 1b. Notably, the two bounds intersect at approximately $A\approx 337.72$^5^.

Let $f(\textbf{x})$ represent one of these boundary functions, where $\textbf{x}$ is an array of variables including *A*, *Z*, and *N* (e.g., $N = A - Z$) and certain selected powers of these variables. Equation (2) describes the general form of a multivariate analytic continued fraction utilized in our work^5^:

2 $$\begin{aligned} f(\textbf{x}) = g_0(\textbf{x})+ \frac{{h_0(\textbf{x})}}{{g_1(\textbf{x})}+ \frac{{h_1(\textbf{x})}}{{g_2(\textbf{x})}+ \frac{{h_2(\textbf{x})}}{{g_3(\textbf{x})}+ \genfrac{}{}{0.0pt}0{}{{\ddots }+ \frac{{h_{n-1}(\textbf{x})}}{{g_n(\textbf{x})}}}}}}, \end{aligned}$$

where *n* indicates the depth of the continued fraction (CF) or $depth\_n$, $g_i(\textbf{x})\in \mathbb {R}$ for all integers $i\ge 0$, each function $g_i:\mathbb {R}^n \rightarrow \mathbb {R}$ is associated with a vector $\mathbf {a_i}\in \mathbb {R}^n$ and a constant $\alpha _i\in \mathbb {R}$, as well as $h_i:\mathbb {R}^n \rightarrow \mathbb {R}$ is associated with a vector $\mathbf {b_i}\in \mathbb {R}^n$ and a constant $\beta _i\in \mathbb {R}$. The total number of variables (*c*) employed in the function and the variables are described in the vector $\textbf{x}=[x_1,\ldots ,x_c]$ with their respective coefficients represented in the vector $\mathbf {a_i}=[a_{i_1},\ldots ,a_{i_c}] \ \mathrm{and} \ \mathbf{b_i}=[b_{i_1},\ldots,b_{i_c}]$. Following previous work^6,9–12^ we have used linear functions in^5^, as follows.

3 $$\begin{aligned} g_i(\textbf{x})= & {} \mathbf {a_i^Tx} +\alpha _i, \end{aligned}$$

4 $$\begin{aligned} h_i(\textbf{x})= & {} \mathbf {b_i^T x}+\beta _i. \end{aligned}$$

As a result, the process of obtaining effective upper and lower bounds becomes a complex optimization problem that combines combinatorial and nonlinear elements. This task involves utilising a given experimental dataset to discover suitable upper and lower bound models, which ultimately depends on identifying the sets of coefficients ${\mathbf {a_i}}$, ${\mathbf {b_i}}$, ${\alpha _i}$, and ${\beta _i}$. While our previous paper is strongly focused on establishing two different mathematical models that act as upper and lower bounds of the nuclear binding energy, this manuscript tackles a significantly more challenging task: approximating the precise value for each entry in the dataset with a single model. To illustrate this concept, consider a recent study where continued fraction regression (cf-r) was employed to derive analytical approximations for the minimum electrostatic energy configuration of *n* electrons, denoted as *E*(*n*)^10^. In the aforementioned study, the electrons were constrained to reside on the surface of a sphere, addressing the solutions of the Thomson Problem^10^.

Our approach differs from other data-driven models aimed at approximating the binding energy per nucleon, which have predominantly relied on Artificial Neural Networks (ANN). For instance, in Ref.^13^, researchers proposed the use of an ANN to model and predict nuclear binding energy across a wide range of proton and neutron numbers, with the objective of identifying the elusive ‘island of stability’. Another example can be found in Ref.^14^, where an ANN model was employed to predict residuals between experimental values obtained from the Atomic Mass Evaluation 2020 (AME2020) report^8^ and values predicted using the Liquid Drop Model (LDM). This ANN aimed to enhance prediction precision.

While methods like ANNs are capable of modelling NBE^13^ or predicting residuals from LDM^14^, they are often regarded as ‘black-box’ models, lacking interpretability. In contrast, this work proposes an alternative approach that leverages data from AME2020 and symbolic regression methods to generate interpretable analytic functions, which are perhaps more amenable to downstream studies via uncertainty propagation and sensitivity analysis and thus more “explainable”^6,7^.

Following^15^ and^16^, there is a renewed interest in the application of symbolic regression methods to the problem of identifying new physical laws and also to model unknown independent variables from experimental data for which they have incomplete knowledge without subjecting human bias^7^.

In our case, we are primarily interested in achieving accurate approximations with low complexity that effectively model the data. To accomplish this, we employ the formal concept of ‘convergents of a continued fraction’, which aligns perfectly with our objective. We can opt to truncate the expansion precisely when each of the convergents is formally defined, allowing us to assess both the fit and complexity of these convergent-based models. The explicit analytic forms of these upper and lower bound models identified in^5^ are provided in Supplementary Material.

### Nuclear stability and the Liquid Drop Model

In the 1930s, two pivotal papers emerged in the field of nuclear physics: one by Weizsäcker^1^ and another by Bethe^2^. These papers gave birth to the LDM, a foundational concept. The LDM envisions the nucleus as a charged, irrotational spherical liquid drop, with its energy comprising several components, including a ‘volume energy’, a ‘surface energy’, and a ‘Coulomb energy’. Additionally, it includes two more specific terms known as the ‘asymmetry’ and ‘pairing’ terms^2,17–20^.

As articulated in^20^, the LDM can be represented as *B*(*A*, *Z*, *N*),

5 $$\begin{aligned} B(A,Z,N)&\approx a_{V} \, A - a_S \, A^{\frac{2}{3}}-a_C \, Z(Z-1) \, A^{-\frac{1}{3}} \nonumber \\&\quad - a_A \, (A-2\,Z)^2 \, A^{-1} + \delta (N,Z)\,A^{-\frac{1}{2}}, \end{aligned}$$

where $$\delta (N,Z)$$ is,

6 $$\begin{aligned} \delta (N,Z) = \delta _0 \, \frac{(-1)^N+(-1)^Z}{2} \,, \end{aligned}$$

known as the *pairing term*. $\delta (N,Z)$ is either zero or $\pm \delta _{0}$ depending on the parity of number of neutrons *N* and protons *Z*^19–22^. The free parameters found in the model derive from least squares to fit experimental data and can be found in^20^; they are the *volume coefficient*, $a_V=15.192$; the *surface coefficient*, $a_S=16.269$; the *Coulomb coefficient*, $a_C=0.679$; the *asymmetry coefficient*, $a_a=21.675$ and the previously mentioned called the *pairing term*, $\delta _0=10.619$. It is known that further terms exist to explain additional phenomena^4,23^ and that this model does not fit well the data for low values of *A* as we will show in our results.

## Databases

### The AME2020 Database

We have used the data obtained from the AME2020^8^ and National Nuclear Data Center Database (NuDat) and it was restricted to the most highly stable nuclides. We now look at unstable nuclides experimentally found that might exhibit new features not present in stable nuclei^24^ as previously mentioned. Seeking to demonstrate the power of our method amid data showing different features, this study explores distinct characteristics through the use of two different datasets explained below. Regardless of the dataset, each sample describes a nuclide by its value of the binding energy per nucleon (in *MeV*) defined as the dependent variable, and by its *Z*, *A* and *N*, defined as the independent variables. To enhance the model’s performance, the original set of inputs is extended using a new set of meta-features, employing the powers *p* with $p \in \left\{ -3, -2, -1, -\frac{2}{3}, -\frac{1}{2}, -\frac{1}{3}, \frac{1}{3}, \frac{1}{2}, \frac{2}{3}, 1,2,3 \right\}$ of the independent variables to create the meta-variables to find models with less complexity (as we did in^25^). We also included the *parity* described in Eq. (6) with $\delta _0=1$. The value $\delta _0=1$ was defined to only represent each nuclide’s parity sign, due to the fact that if we analyse Eqs. (3) and (4), it is possible to notice that if the parity term is included in a function employed in a model the contribution of parity will be weighted by the coefficients found by the algorithm.

### Dataset 1—Tritium and 254 most stable nuclides from AME2020 also used in Ref.^22^

According to IAEA, an isotope is considered *stable* when it is non-radioactive. The Nuclear Data Services provided by IAEA (International Atomic Energy Agency) defines 243 stable isotopes in the Nuclear Chart (see Supplementary Material). However, in^22^, the authors used a reduced number of nuclides in their study, a total of 109 nuclides (see Supplementary Material). They used 96 stable isotopes and included 12 long-lived isotopes and the tritium in their selection of stable isotopes, all inclusions are described and their measured or estimated half-lives are indicated in Supplementary Material.

To help with further comparisons we opted to select the isotopes from^22^ for the training subset due to the reduced number of elements, allowing us to demonstrate the performance of our method even when using a smaller number of observations. This training subset was the one used in the first example of symbolic regression by applying the Thomson problem to find an alternative numerical approximation of the binding energy per nucleon for highly stable nuclei.

Aiming to demonstrate how our method would perform when confronted with the remaining stable isotopes from the nuclear chart, the testing subset contains the other 147 stable isotopes.

### Dataset 2—2531 experimentally observed values of AME2020

This dataset comprises a total of 3535 nuclides with $A \ge 8$ for the stable and unstable nuclides. From these 3535 nuclides, 2531 had their binding energy measured and the remaining 1004 nuclides had their binding energy estimated^8,26^. Based on this aspect we defined the nuclides with experimentally observed values of binding energy as the training subset, represented by red crosses in Fig. 1a. Whereas the nuclides with an estimated value of binding energy as the testing subset, represented in Fig. 1b by black crosses.

## Results

We started first the investigation by testing if it would be possible that the putative optimal solutions of the Thomson problem could be leveraged somehow to obtain better approximations. We note that at the time of the publication of the contributions of Weizsäcker^1^ and Bethe^2^ these solutions were not available and the Coulomb term reflects an asymptotic behaviour of a set of classical point charges. We cover this in the following subsection.

### The Thomson problem and an alternative numerical approximation of the binding energy per nucleon for highly stable nuclei

At the beginning of the $20{th}$ century, J. J. Thomson proposed the earliest model of the atom based on his previous discovery of the negatively charged electron. We now know as the Thomson Problem the task of determining the minimum electrostatic potential energy configuration of a number of electrons when they are constrained to the surface of a unit sphere, where each other will repeal with a force given by Coulomb’s law^27,28^. In what follows, without losing generality, we will assume a unit of one for the charges and for Coulomb’s constant. Then the total electrostatic potential energy *U*(*Z*) of a configuration of *Z* protons is proportional to the sum of all pair-wise interactions so we can write,

7 $$\begin{aligned} U(Z) = \sum _{i<j} \frac{1}{r_{ij}}, \end{aligned}$$

where $r_{ij} = |\mathbf {r_i} - \mathbf {r_j}|$ is the distance between each pair of protons located at points on the sphere defined by vectors $\mathbf {r_i}$ and $\mathbf {r_j}$. Then we define *T*(*Z*) as the value of the normalized electrostatic interaction energy occurring between each pair of protons of equal charges in the configuration of minimum energy cost when they are on a sphere of unit radius, so $T(Z) = 2 \, U(Z) Z^{-2}$.

Unlike the early 20*th* century when LDM was developed, we now possess knowledge of *T*(*Z*), thanks to the efforts of researchers over the past four decades. These researchers have employed optimization methods, resulting in putative optimal solutions to the Thomson problem for cases where $Z<200$^10^.

Motivated by this wealth of data, we embarked on an investigation to determine if these values could be leveraged to create an alternative approximation formula to the LDM, potentially offering improved accuracy, especially for $Z<20$. To explore this possibility, we utilized the commercial package TuringBot (https://turingbotsoftware.com/), employing variables such as *A*, *N*, *Z*, and the values of *T*(*Z*) from the putative optimal solutions to the Thomson Problem.

The package TuringBot was used without any special parameter fine-tuning, but indeed some selections are obviously needed. For instance, with some symbolic regression packages, you often need to specify the mathematical functions to use. We only employed the four basic arithmetic functions (addition, subtraction, multiplication, and division). The coefficients were chosen to be integers and the objective function to minimize was the Mean Squared Error.

Our expectation was that, as symbolic regression methods inherently aim for lower model complexity, TuringBot might rediscover the equation of the LDM or provide an alternative one. However, employing data from AME2020^8^, we obtained a new and intriguing approximation, as demonstrated by Eq. (8).

8 $$\begin{aligned} \frac{B(A,Z)}{A}\approx \left( \frac{A^2 - 1221 \,A + 6350}{164 \, (5 - A)}\right) \left( \frac{Z}{A}+ T(Z) \right) . \end{aligned}$$

In a second experiment, we included the parity of *N* and *Z* (with $\delta _0 = 1/(2A)$.) as a variable and we obtained Eq. (9) which again has the same functional form.

9 $$\begin{aligned} \frac{B(A,Z)}{A} \approx \left( \frac{A^2 - 1229 \, A + 6289}{166 \, (5 - A)} \right) \, \left( \frac{Z}{A}+ T(Z) \right) \, \left( 1+\delta \left( N,Z\right) \right) \end{aligned}$$

**Figure 2:**
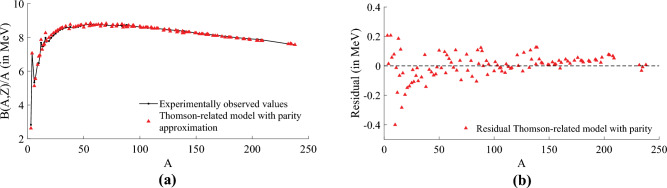
Approximation of the stable and long-lived nuclides of the training subset of Dataset 1 using the data-driven Thomson-based model represented in Eq. (9) and the residual for the experimentally observed values (data from AME2020^8^) of the training subset of Dataset 1 containing stable and long-lived isotopes (109, for same nuclei of Ref.^22^). (**a**) NBE per nucleon according to Eq. (9) and comparison with the experimental values (black). (**b**) Residual of data-driven approximation by symbolic regression that delivered Eq. (9).

**Figure 3:**
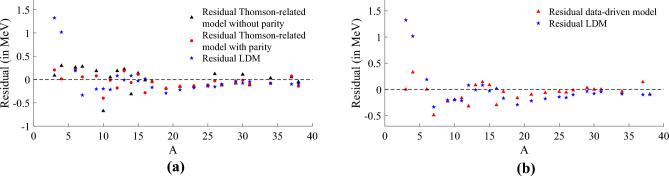
Residual of approximations of the stable and long-lived nuclides of the training subset of Dataset 1 (see “Databases” section) restricted to $A<40$. In (**a**), the residual of approximations using the Thomson data-driven model with parity (red) and without parity (black), described in Eqs. (9) and (8) respectively. Both models are compared with LDM (blue), where the model using parity demonstrates a better approximation capability, particularly for lighter nuclides, than the model without parity and LDM. In (**b**), the residual of approximations using the data-driven model from Eq. (10) (red) and the LDM (blue). The data-driven model approximations are more accurate and outperform LDM for nuclides with $N\le 20$, highlighting the first and third approximations showing smaller errors in the order of $10^{-6}$, this efficiency repeats for elements at $A\approx 30$.

The functional form of Eqs. (8) and (9) surprised us in some sense. For instance, Eq. (9) is proportional to the parity, another term that is proportional to the ratio of protons to nucleons plus *T*(*Z*) and a third term that is a ratio of two low-order polynomials on *A* only. This term has a pole for $A=5$ and for this value, no nucleus with $A=5$ has been found. The Mean Square Error (MSE) of Eq. (8) is $1.150\times 10^{-2}$, contrasting with $MSE=7.856\times 10^{-2}$ for the LDM and an $MSE=7.498\times 10^{-3}$ when parity is included in Eq. (9). Figure 2a represents the approximation using Eq. (9) and its respective residual in Fig. 2b. Most interestingly, both Eqs. (8) and (9) are better approximations than the LDM, particularly for small *A* as Fig. 3a shows.

We should also note our previous result in^5^ which we found that

10 $$\begin{aligned} \frac{B(Z,N)}{A} \approx c_0 + \frac{c_1}{\sqrt{Z}} + c_2 \, \sqrt{N} + c_3 \, \sqrt{Z} + \frac{\delta (N,Z)}{A} \,, \end{aligned}$$

where $c_0=10.79546163$, $c_1=-7.611300946$, $c_2=-0.700133274$, $c_3=0.633240196$ and $\delta (N,Z)$ is the parity term using $\delta _0 = 5.7175$. Comparing this approximation’s MSE of $1.103\times 10^{-2}$ with the LDM, it is also a better approximation (residual represented in Fig. 3b). Together this indicates that it may be possible to approximate well using a truncated analytic CF of small depth. This has motivated us to find numerical approximations of the nuclear binding energy directly using the CF representation and the rest of our work helps to extend the approach to all nuclides.

## A data-driven approximation for *B*(*A*, *N*, *Z*)/*A* for all nuclides based on continued fractions

Up to this point in our paper, our focus has primarily centered on approximating stable and long-lived nuclides. Nevertheless, a significant challenge in modern nuclear physics lies in the exploration of unstable nuclides, particularly those at the limits of stability^29^. Investigating these unstable nuclei offers a profound understanding of nuclear interactions and unveils novel features not present in stable nuclei. The quest to derive the properties of atomic nuclei from the interactions among their constituent nucleons has long been a central pursuit in nuclear physics^24,30^.

A pivotal realm of research delves into the heaviest nuclides on the nuclear chart, commonly referred to as superheavy nuclides (SHN), characterized by $Z \ge 104$ in the transactinide region. All currently known SHN are radioactive and have been synthesized through nuclear reactions by dedicated scientists^30–33^. The study of SHN is essential for addressing fundamental questions such as ‘How are superheavy nuclei and atoms formed and organized?’, ‘Do extraordinarily long-lived superheavy nuclei exist in nature?’, and ‘What are the heaviest nuclei that can naturally exist, and where does the Periodic Table of Elements ultimately conclude?’^30^.

### Identification of model using continued fraction regression via memetic algorithms

In our efforts to optimize the models generated using cf-r, we employed a Memetic Algorithm (MA), a population-based meta-heuristic technique, for variable selection and coefficient determination. MAs were initially introduced by one of the authors in 1989^34^ and, in this context, serve as the chosen evolutionary technique. They are applied to fine-tune both the selection of parameters and variables within the formula, aiming to identify the fittest model based on the designated loss function. This procedure plays a crucial role in enhancing the regressor’s performance, and its significance is further elucidated in our paper.

The MA framework encompasses evolutionary processes that incorporate mutation and recombination procedures, along with a local search algorithm that operates within the generation cycles to tackle complex problems^35,36^.

In this contribution we are using a modified version of the Nelder-Mead proposed in^37^ for its simplicity and quality. It has been used in our methods since 2018 because it allows us to experiment with many types of objective functions. The modified NM version operates using a simplex defined by $(n+1)$ vertices and a centroid, all obtained from a single initial solution provided. The algorithm manipulates the simplex vertices in each iteration by replacing the worst vertex with a better one. In each iteration, all vertices are classified according to their loss function value. This variant simplifies the NM method by eliminating redundant elements while retaining the fundamental set of terminals and functions, ultimately resulting in a tree-structured representation^37^.

To facilitate parameter and variable selection within the MA, we defined the following parameters: a mutation rate of 0.1, a feature selection parameter (referred to as $\Delta$) set to 0.1, and a total of 200 generations. The chosen MA structure is a depth-3 ternary tree-based population structure. The NM method uses one initial solution, as mentioned before, and a total of 250 generations are performed along 4 runs of the local-search in each generation of the MA. It is worth noting that these parameters remain consistent throughout all experiments conducted and were derived from prior work^9,11^. To evaluate the fitness of the obtained solutions, we employ the MSE metric. This metric, combined with the feature selection parameter $\Delta$, forms our defined loss function, denoted as $\ell = MSE \times (1 + \Delta \times \# \text { of features})$.

Our initial task was to determine the appropriate CF depth for cf-r that best fits the data. To achieve this, we aimed to minimize the MSE of the fit, irrespective of the model’s depth (complexity). We pursued this by conducting 25 independent runs of the MA, starting from $depth_0$, for a given dataset. At each depth, we selected the best-performing model. We terminated this process when we observed that the MSE of the selected model ceased to decrease with increasing depth. This signified that the preceding depth had returned a model with a lower MSE and lower complexity than subsequent runs. Following the selection of the CF depth using this approach, we executed another 100 independent runs to identify the best model, all at the same selected depth. These additional runs provided us with further insights into the performance of the MA and its consistency in discovering models of similar quality. We also present a descriptive statistical analysis of the results for both datasets in Supplementary Material.

All cf-r models presented in this study utilize real-number coefficients, approximated to rational numbers with a precision of 13 digits. This level of precision is necessary to prevent performance degradation. The rational approximations are generated using the MATLAB function rats, which truncates continued fraction expansions to obtain the rational approximation with the specified precision. The CF coefficients are derived by iteratively extracting the integer part of the real number and then calculating the reciprocal of the fractional part.

To identify the most reliable model, we divided the process into a training phase using the training subset and a testing phase using the testing subset data available in Dataset 1 (as described in “Databases” section). To enhance statistical robustness, we conducted a 10-fold cross-validation with 100 runs per fold. Detailed results and statistical analyses are provided in the Supplementary Material.

Dataset 2 served as a valuable resource for assessing the predictive quality of models that exclusively use experimentally confirmed values as their training set. These models were employed to predict the 1001 nuclear binding energy (NBE) values of AME2020.

Moreover, Dataset 2 facilitated another experiment of particular interest. We aimed to derive a model that excelled in predicting NBE values for nuclei with $A\ge 200$ while still maintaining strong performance across the entire Dataset 2. Instead of selecting the best model based solely on the overall performance on Dataset 2 (in terms of Mean Squared Error, MSE), we chose the model in the 100 independent runs that demonstrated the best fit (lowest MSE) for all nuclides with $A\ge 200$. Additionally, we conducted a 10-fold cross-validation using only the experimentally observed values to assess the statistical robustness of our approach.

### Results on the different datasets

In this section, we present the results obtained using cf-r with both datasets mentioned earlier. Initially, we analyze the results derived from Dataset 1, which comprises the 109 most stable and long-lived nuclides sourced from AME2020. This dataset was also used in a prior study (Ref.^22^).

Next, we delve into the results generated using Dataset 2, which consists of 2531 experimentally observed nuclides, as discussed in “Databases” section. We also provide a detailed analysis of results with a focus on nuclides with $A\ge 200$. Lastly, we explore the model’s extrapolation capability to predict NBE in the range of $296\le A \le 338$.

### For Tritium and 254 most stable nuclides (Dataset 1)

**Figure 4:**
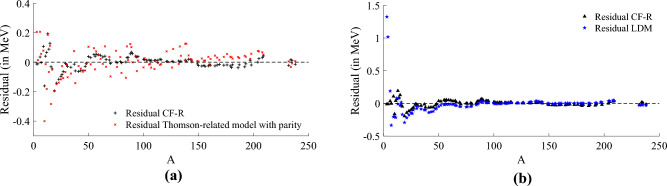
Both plots show the residual of the approximations for the experimentally observed values of the training subset of Dataset 1 which contains a reduced number of stable and long-lived nuclides from AME2020. Residuals of the approximation using cf-r given by Eq. (12) compared to the Thomson-related model with parity (Eq. (9)) in (**a**), and later compared with LDM (Eq. (5)) in (**b**). The cf-r model shows a better approximation if compared to either the Thomson-related model with parity or LDM, achieving smaller residuals, especially for the lighter nuclides.

According to the methodology and parameters setting previously described for the experiment, we found that a $depth_1$ CF expressed below in Eq. (11),

11 $$\begin{aligned} \frac{B(Z,N)}{A} \approx g_0(Z,N)+\frac{{h_0(Z,N)}}{{g_1(Z,N)}}\,, \end{aligned}$$

is capable of approximating the NBE of the experimentally observed values of the training subset of Dataset 1 which contains a reduced number of stable and long-lived nuclides from AME2020. In an attempt to enhance our model’s approximation, we performed an extra optimisation step to improve only the model’s coefficients and constants. This optimisation was done using the well-known multivariate Newton’s method^38–40^ (more information in Supplementary Material). The best model achieved after the optimisation reduced the MSE in $10.45\%$, from $2.805\times 10^{-3}$ to $2.519\times 10^{-3}$. The optimised model is described below,

12 $$\begin{aligned} g_0(Z,N)&= \frac{61}{101430}\,\delta (N,Z) -\frac{118}{63203}\,Z^{\frac{3}{2}} -\frac{508}{16161}, \nonumber \\ h_0(Z,N)&= \frac{2917}{5655}\,\delta (N,Z) +\frac{2745}{3877}\,Z^{\frac{3}{2}} - \frac{3401}{13936},\nonumber \\ g_1(Z,N)&= \frac{242}{12889}\,\delta (N,Z) +\frac{281}{3694}\,Z^{\frac{3}{2}} -\frac{1922}{3777}\,N^{-1} +\frac{1315}{3869}, \end{aligned}$$

where $N=A-Z$. The model obtained also employs the *parity* described in Eq. (6), with $\delta _0=1$. The residual of the approximation using Eq. (12) is presented in Fig. 4, where cf-r is compared to the Thomson-related model with parity (Eq. (9)) in Fig. 4a, and also compared with LDM (Eq. (5)) in Fig. 4b.

The cf-r model shows a better approximation and achieves smaller residuals, especially for the lighter nuclides, if compared to either the Thomson-related model with parity or LDM.

### For 2531 experimentally observed NBE values (Dataset 2)

In our pursuit of obtaining the most reliable model, we adopted a two-phase strategy. During the training phase, we utilized empirical data, while the testing phase involved evaluating the model’s performance against estimated data, as described in the subsets explanation. Once again, we found that a $depth_1$ CF model expressed in Eq. (11) yielded the most effective data-driven approximation for the problem.

Upon analyzing the results of 100 runs, we noted that most models performed better when approximating heavier nuclides and less effectively when dealing with lighter nuclides. To address this observation, we introduce a weighting mechanism for each nuclide in the dataset. This weight acts as a penalization factor when calculating the loss function, encouraging the algorithm to obtain better solutions for observations with higher weight values assigned. The loss function with this penalization factor, denoted as $\ell _{Pen}$, is calculated as $\ell _{Pen} = (err^2 + \frac{1}{A} \times k + 1)^4$, where we empirically set *k* to 50.

The introduction of the $\ell _{Pen}$ loss function resulted in a notable improvement in the model’s ability to approximate lighter nuclides. Additionally, it led to a reduction in the MSE for both experimentally observed values (training subset) and estimated values (testing subset).

**Figure 5:**
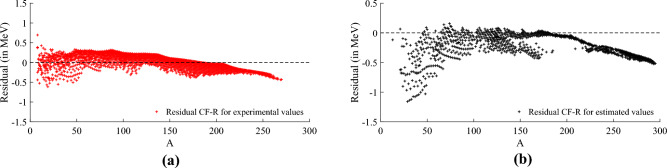
Prediction residual of the best model found represented in Eq. (13) using cf-r for experimentally observed values of stable and unstable nuclides (red) used in the training phase and estimated values of unstable nuclides (black) used in the testing phase. It is possible to notice that the absolute residual for most of the experimentally observed values is smaller than 0.5 MeV, whilst the approximation for the estimated values generated larger residuals mostly for nuclides with $A<75$. (**a**) Residual of experimentally observed values (training subset). (**b**) Residual of estimated values (testing subset).

Furthermore, the maximum absolute residual, a significant metric, decreased from approximately 1.5 MeV to around 0.5 MeV in the training subset and from roughly 2 MeV to about 1.25 MeV in the testing subset. A more comprehensive statistical analysis of these experiments is available in the Supplementary Material. The best model achieved is expressed below,

13 $$\begin{aligned} g_0(Z,N)&= -\frac{367}{44248}\,A^{-2} -\frac{445}{68681}\,A^{-\frac{2}{3}} +\frac{773}{59640}\,Z^{-2} +\frac{38}{971}\,N^{-2} - \frac{1711}{165801},\nonumber \\ h_0(Z,N)&= \frac{1123}{43516}\,A^{-2}+\frac{436}{115083}\,A^{-\frac{2}{3}} +\frac{341}{51764}\,Z^{-2} +\frac{130}{100383}\,N^{-2}+\frac{11}{2768605},\nonumber \\ g_1(Z,N)&= -\frac{1429}{84370}\,A^{-2}+\frac{89}{231107}\,A^{-\frac{2}{3}}+\frac{211}{56774}\,Z^{-2}+\frac{81}{24256}\,N^{-2}+\frac{3}{999001}, \end{aligned}$$

and the prediction residual of this model can be observed in Fig. 5, where Fig. 5a shows the residual for the approximation of the experimentally observed values of NBE and Fig. 5b shows the residual for the approximation of the estimated values of NBE. We highlight the maximum absolute residual for most of the approximations of the experimentally observed values are smaller than 0.5 MeV, while the approximation for the estimated values produced larger residuals mostly for nuclides with $A<75$. This can be explained by the fact that most of the nuclides with estimated NBE values are located below the nuclides with experimentally observed NBE values or in the area known as SHN region, as shown in Fig. 1, turning the approximation task more difficult for these nuclides.

**Figure 6:**
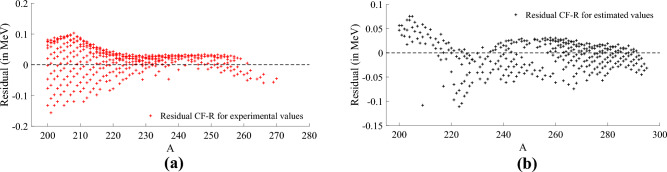
Prediction residual of the best model for the experimentally observed values (red) and estimated values (black) of Dataset 2 considering nuclides with $A\ge 200$. It is possible to verify that the model expressed in Eq. (14) predicts the binding energy of unstable nuclides with an absolute residual smaller than approximately 0.15 MeV for both experimentally observed values (training subset) and estimated values (testing subset). The heaviest experimentally observed value is at $A=270$, demonstrating the good extrapolation performance of our model for heavier nuclides with $A>270$ and only estimated values of NBE. (**a**) Residual of the model (described in Eq. (14)) prediction of the experimentally observed values (red) considering *A* ≥ 200. (**b**) Residual of the model (described in Eq. (14)) prediction of the estimated values (black) considering *A* ≥ 200.

### Best model for nuclides with $A\ge 200$

Due to the enhanced approximation capabilities of cf-r for heavier nuclides, we have undertaken an evaluation to identify the best model from among the 100 runs conducted, specifically focusing on cases where $A\ge 200$. The optimal approximation is represented by the convergents detailed in Eqs. (14) below,

14 $$\begin{aligned} g_0(Z,N)&= -\frac{219}{28834}\,A-\frac{518}{8595}\,Z^{-\frac{2}{3}}-\frac{1192}{31983}, \nonumber \\ h_0(Z,N)&= \frac{944}{53413}\,A+\frac{359}{2388}\,Z^{-\frac{2}{3}}+\frac{1676}{10511},\nonumber \\ g_1(Z,N)&= \frac{149}{78640}\,A+\frac{921}{7619}\,Z^{-\frac{2}{3}}+\frac{301}{33242}, \end{aligned}$$

by substituting Eqs. (14) into Eq. (11), we derive the model. Its performance in approximating NBE for $A\ge 200$ can be observed in Fig. 6. Specifically, Fig. 6a and b display the prediction residuals for the experimentally observed (training subset) and estimated values (testing subset) of NBE, respectively. In both plots, it is evident that the model’s absolute residuals are consistently smaller than approximately 0.15 MeV for the majority of the nuclides. A detailed statistical analysis of the 100 runs conducted for heavier nuclides ($A\ge 200$) can be found in Supplementary Material.

### Extrapolation results of Eq. (14) for $A>295$

The heaviest element that has been synthesized to date possesses an atomic mass of $A=295$, with $Z=118$ and $N=177$ (as reported in^8^). There is substantial evidence suggesting that elements with $Z\ge 100$, belonging to the SHN region, exhibit a pronounced dependence on the shell structure of both protons and neutrons. This dependence is closely related to the concept of ‘magic numbers’, which are specific values for the numbers of protons and neutrons that enhance the stability of nuclei against spontaneous fission.

Recent research, as cited in^41–43^, has identified predicted proton magic numbers for elements with $Z\ge 82$ as 82, 98, 100, 102, 106, 108, 114, 116, 120, and 126. Similarly, predicted neutron magic numbers for elements with $N\ge 126$ include 126, 148, 152, 154, 160, 162, 172, 176, 178, 180, 182, 184, and 200. It is anticipated that nuclei with nucleon count closely matching these magic numbers exhibit heightened stability.

**Figure 7:**
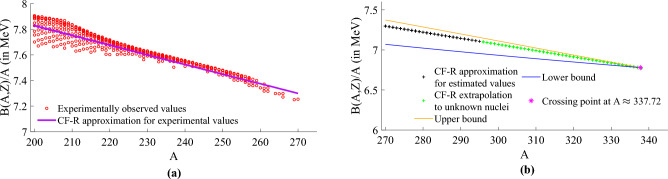
The left plot illustrates the behaviour of the model described in Eq. (14) when exploring the upper limits of nuclear mass in the range of $200\le A\le 270$ (purple). The right plot represents the extrapolation of our model to the unknown upper limits of nuclear mass in the range of $296\le A\le 338$ (green). We included the upper (orange) and lower (blue) bounds from Ref.^5^ (given in Supplementary Material) for $A\le 338$ to demonstrate that the model analysed behaves in agreement with the prediction of the lower and upper bounds when extrapolated. In the range of $200\le A \le 295$, the approximations from Eq. (14) for the experimentally observed values of NBE (red) and for the estimated values of NBE (black) are included. It is possible to verify that the model converges to the meeting point (pink) of the upper and lower bounds.

As previously demonstrated, our data-driven technique provides both lower and upper analytical bounds to *B*(*A*, *Z*) (see Supplementary Material), with the two bounds converging at $A\approx 337.72$^5^. To assess whether the model described in Eq. (14) aligns with the predictions of these lower and upper bounds, we conducted an extrapolation study to explore the upper limits of nuclear mass. To perform this extrapolation, we leveraged Eq. (14), which requires both the atomic mass and atomic number. We selected values for the atomic number within the range of $114\le Z \le 126$ from the sequence of proton magic numbers mentioned earlier. This allowed us to create plausible combinations of protons and neutrons, thereby assessing the model’s extrapolation capability in the range of $296\le A \le 338$. The results, as depicted in Fig. 7, confirm that the out-of-domain predictions using Eq. (14) converge to the point where the upper and lower bounds intersect, occurring at $A\approx 337.72$.

## Conclusion

In this study, we introduced a data-driven approach, Continued Fraction Regression (cf-r), to model nuclear binding energy (*B*(*A*, *Z*)) for atomic nuclei. Our method, equipped with a customized loss function and an analytic continued fraction representation, has proven to be a powerful tool for approximating nuclear binding energies with remarkable precision. One of the significant contributions of our work is the comparison of our data-driven model with traditional models like the Liquid Drop Model and the exploration of its applicability for a wide range of nuclides. Furthermore, the value of our models does not lie in quantitative calculations of nuclear binding energies, but lies, in our opinion, in qualitative results comparable with traditional models. We might try to deduce the dependence of the nuclear binding energy on the nucleonic properties, like—to a first degree of approximation—the number of neutrons and protons. Still, all these conclusions should be considered tentative only. Notably, we outperformed the Liquid Drop Model, particularly excelling in the regime of nuclides with $A<20$. This underscores the potential of symbolic and continued fraction regression as an alternative representation for machine learning problems. Our model’s versatility and accuracy are further highlighted by its ability to provide precise predictions for nuclides with $A\ge 200$, achieving residuals consistently below 0.15 MeV. This capability has implications for a myriad of nuclear physics studies, including the investigation of superheavy elements and the determination of magic numbers. Moreover, we demonstrated the extrapolation potential of our model, showcasing its convergence with upper and lower bounds as it approaches the nuclear mass limit. This validates the accuracy and robustness of our approach and opens new avenues for exploring nuclear properties beyond the known boundaries.

The wide array of nuclides, including not only the limited set of roughly 300 stable ones but also approximately 6000 to 7000 partially or entirely unknown exotic nuclides awaiting synthesis and exploration, offers fertile ground for the emergence of new physical phenomena, particularly within specific regions of the nuclide chart. These phenomena include halo nuclei (e.g., $^{11}_{3} \text {Li}$), neutron-rich nuclei located near the neutron drip line, and groundbreaking giant resonance modes observed in heavy nuclei, among others. Similarly, regions neighbouring the proton drip line follow a similar pattern. Therefore, we fully acknowledge the difficulty in endorsing the notion that a data-driven method of approximation, without regard for underlying physical phenomena, can provide accurate assessments of the extreme regions of the nuclide chart based solely on available experimental and estimated data. Additionally, the incorporation of estimated nuclides does not reveal the novel physical phenomena unique to exotic regions. It is essential to understand that data-driven machine learning compresses data and does not imply the absence of new physics or confine experimental values of *B*(*Z*/*N*) within predetermined bounds. Thus, from a physical modelling perspective, the advancement of a highly precise approximation of the binding energy per nucleon (*B*/*A*) as a function of proton number (*Z*) and neutron number (*N*) can enrich our understanding of nuclear forces only if each term in the proposed approximation corresponds to a distinct physical phenomenon.

In conclusion, our data-driven methodology offers an innovative way to model nuclear binding energies, complementing traditional approaches and holding promise for various applications in nuclear physics, as we continue to refine and expand it.

## Supplementary Information

Supplementary Information.

## Data Availability

The datasets generated during and/or analyzed during the current study are available in the public domain and was published in the AME2020^8^, available at https://www-nds.iaea.org/amdc/ame2020/mass_1.mas20.txt, and from the National Nuclear Data Center Database (NuDat), available at https://www.nndc.bnl.gov/nudat3/. We have made publicly available the datasets employed in the TuringBot software, Matlab files containing all equations detailed in this study, and the corresponding plots, as well as spreadsheets with these equations and plots. These can be accessed at https://figshare.com/projects/Approximating_the_Nuclear_Binding_Energy_Using_Analytic_Continued_Fractions/202593.
