# Supplementary material for: Approximating the nuclear binding energy using analytic continued fractions
Source: Sci Rep. 2024 May 21;14:11559. doi: 10.1038/s41598-024-61389-5 (PMC11636920; doi:10.1038/s41598-024-61389-5)
Supplement: Supplementary file 1 — Supplementary Information. [file 41598_2024_61389_MOESM1_ESM.pdf]

Supplementary Material

Upper and lower boundaries models

The upper and lower boundary models developed using the continued fraction method are expressed below, where A is the atomic mass number. The general form of a bound CF is expressed as,

$$\left(\frac{B(A)}{A}\right)_X \approx g_0(A)_X + \frac{h_0(A)_X}{g_1(A)_X}, \tag{S1}$$

where $X \in \{LB, UB\}$ is one of two possible labels indicating if we are referring to a lower or upper bound (respectively LB, UB). Accordingly, for the upper bound we have Supplementary Eqs. (S2) below:

$$\begin{aligned}
g_0(A)_{UB} &= \frac{1481}{158} - \frac{1}{109}A, \\
h_0(A)_{UB} &= \frac{54369}{148} - \frac{873}{134}A, \\
g_1(A)_{UB} &= -\frac{16351}{308} - \frac{20914}{1973}A,
\end{aligned} \tag{S2}$$

and for the lower bound Supplementary Eqs. (S3):

$$\begin{aligned}
g_0(A)_{LB} &= \frac{451}{10740}A^{\frac{1}{3}} - \frac{871}{11709}A^{-\frac{1}{3}} + \frac{25}{1092}, \\
h_0(A)_{LB} &= \frac{70}{4699}A^{\frac{1}{3}} + \frac{322}{21897}A^{-\frac{1}{3}} - \frac{419}{45192}, \\
g_1(A)_{LB} &= \frac{17}{3278}A^{\frac{1}{3}} + \frac{428}{5699}A^{-\frac{1}{3}} - \frac{79}{2470}.
\end{aligned} \tag{S3}$$

Utilizing TuringBot

When utilizing TuringBot, one must define a target and specify a set of input variables. In the discovery of Eq. (8), we selected B/A as the target variable, along with N, A, Z, and $T(Z)$ as input variables, incorporating basic arithmetic operations such as addition, subtraction, multiplication, and division. We restricted coefficients to integers within the formulas. For Eq. (9), we introduced a parity term $\delta(N,Z)$ as outlined in the text.

The selection of models was guided by the *Root Mean Squared Error* as a loss function, utilising all available entries to generate the models represented by Eqs. (8) and (9). Additionally, default parameters were maintained for certain settings within TuringBot's *advanced window* option, including *maximum formula size* and *maximum history size*, set to 60 and 20 respectively. All other options remained deactivated, consistent with the default configurations.

Empirical approximation for $T(Z)$

Additionally, an empirical approximation for $T(Z)$ is given here. We can denote it as $T_a(Z)$ and it is given by,

$$T_a(Z) = k^{\gamma(Z)}, \tag{S4}$$

where $k = 11591/547$ is a constant and $\gamma(Z) = 4/(6 - 11\sqrt{Z})$. This expression was first found by one of the authors using the academically publicly available symbolic regression package Eureqa in early 2020, further improved by non-linear optimization, and it is also discussed in[10]. It was found using the ansatz of a possible simple dependence of $T(Z)$ on the square root of the number of electrons, which finally led to a mathematical expression with significantly low complexity. See[10] for other approximations.

If instead of the tabulated data for the optimal solution of the Thomson Problem an analytical formula is needed, we have recomputed the value of $f(A)$ and we produced the following approximation,

$$\frac{B(Z,A)}{A} \approx (1 + \delta(N,Z)) \left(\frac{A^2 - 1336A + 6800}{182\,(5 - A)} \right) \left(\frac{Z}{A} + T_a(Z) \right), \tag{S5}$$

again with $\delta_0 = 1/(2A)$.

Results Dataset 1 testing subset

In this section, we present the results for the Dataset 1 testing subset considering all nuclides and also for $A \geq 8$ due to specific features of lighter nuclides, like deuterium and $^{1}_{2}$He (both included in the Dataset 1 testing subset).

Lighter nuclides, notably isotopes such as $^{2}_{1}$H (deuterium), $^{3}_{1}$H (tritium), $^{3}_{2}$He, and $^{4}_{2}$He, exhibit distinct physical characteristics and strongly varying nuclear binding energies, making them hard to approximate[2–4].

Supplementary Table S1 compares the MSE of LDM and 4 different models proposed in this work and in Ref.[5] for the training task and for the testing task, where both the complete and the restricted subset ($A \geq 8$) are informed. Supplementary Figure S1 shows the residuals of the models included in Supplementary Table S1.

It is possible to verify that `cf-r` model had the best result for the training subset, but it demonstrates a poor performance for the complete testing subset that includes deuterium and $^{1}_{2}$He, more specifically for the $^{1}_{2}$He whose approximation absolute residual is 35.48. For the restricted testing subset, the `cf-r` model approximates as well as LDM, where LDM's MSE is 1.5×10^{-5} better than `cf-r` model's MSE. The approximation from both models and also the approximation from the data-driven model proposed in Ref.[5] can be observed through the representation of the residuals (in MeV) in Supplementary Figure S1a, whilst Supplementary Figure S1b shows the residuals of the approximation of LDM and both Thomson-related (with and without parity) models.

Exploring the results of `cf-r` obtained throughout all 100 runs, Supplementary Fig. S2 represents the average residual (in MeV) and the standard deviation for each nuclide in the training subset (see Supplementary Fig. S2a) and testing subset with $A \geq 8$ (see Supplementary Fig. S2b). Supplementary Figure S2a illustrates the difficult task of approximating the lighter nuclides through the larger values of the standard deviation of these nuclides. This is verified by the deuterium and $^{1}_{2}$He in the testing subset, where the average value was 2.354 and 1.914, and the standard deviation was 11.834 and 27.970, respectively.

MSE	LDM Eq. (5)	Thomson-related Eq. (8)	Thomson-related Eq. (9)	Data-driven Eq. (10)	`cf-r` Eq. (12)
Training	3.180×10^{-2}	1.150×10^{-2}	7.498×10^{-3}	1.103×10^{-2}	2.519×10^{-3}
Testing	6.079×10^{-2}	2.364×10^{-1}	1.848×10^{-1}	8.822×10^{-2}	8.569
Testing*	2.268×10^{-3}	1.330×10^{-2}	1.738×10^{-2}	2.083×10^{-2}	2.283×10^{-3}

* $A \geq 8$, deuterium and $^{1}_{2}$He not included.

Supplementary Table S1. Comparison in terms of MSE for the approximations of the 109 stable and long-lived nuclides (Dataset 1 training subset) and the remaining 145 stable nuclides of the nuclear chart (Dataset 1 testing subset). We compare the performances using LDM (Eq. (5)), Thomson-related without parity (Eq. (8)), Thomson-related with parity (Eq. (9)), data-driven model (Eq. (10)) and `cf-r` (Eq. (12)). After analysing the residuals, we identified that deuterium and $^{1}_{2}$He proved to be challenging to approximate by all models, based on this we also present the MSE for the testing set restricted to $A \geq 8$, excluding deuterium and $^{1}_{2}$He.

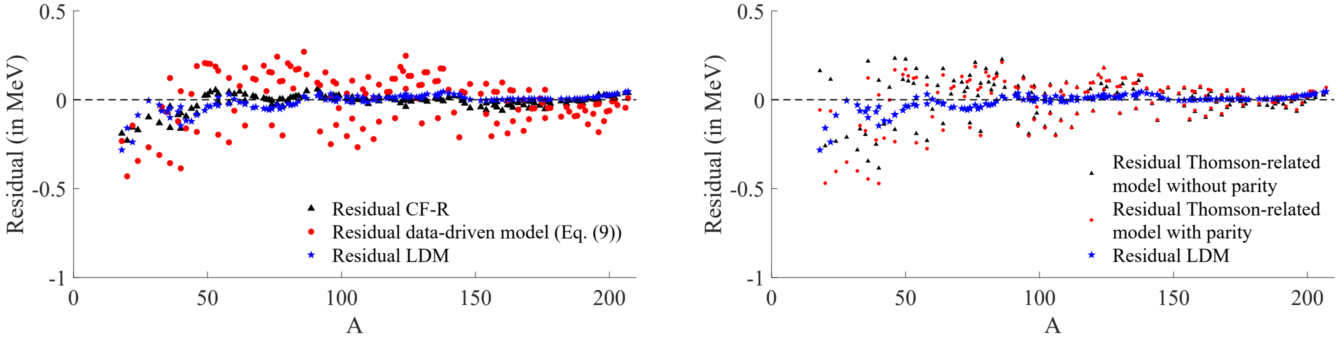

(a) Residuals of the `cf-r` model (Eq. (12)), the data-driven model represented in Eq. (10), and LDM (Eq. (5)).

(b) Residuals of the Thomson-related models without parity (Eq. (8)) and with parity (Eq. (9)), and LDM (Eq. (5)).

Supplementary Figure S1. Residuals for the approximation of the 145 stable nuclides of the nuclear chart not included in the training phase with $A \geq 8$ to improve visualization. We exclude deuterium and $^{1}_{2}$He due to their elevated residual in all models evaluated. The plots show the residual using LDM (Eq. (5)), the Thomson-related models without parity (Eq. (8)) and with parity (Eq. (9)), the data-driven model represented in Eq. (10), and the `cf-r` model (Eq. (12)). We highlight the good approximation obtained with `cf-r` (see Supplementary Fig. S1a), comparable with LDM for lighter and heavier nuclides.

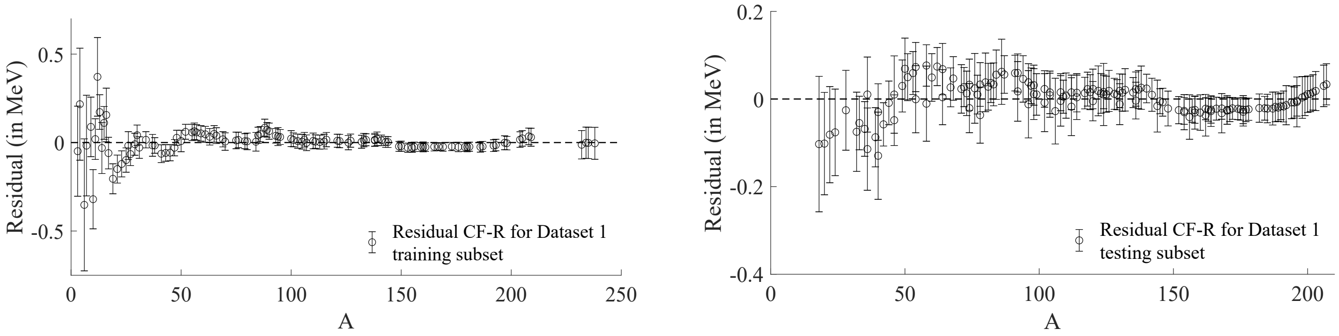

(a) Average residual and standard deviation of 109 stable and long-lived nuclides included the Dataset 1 training subset.

(b) Average residual and standard deviation of 145 stable nuclides with $A \geq 8$ included in the Dataset 1 testing subset.

Supplementary Figure S2. Average residuals for each nuclide and its respective standard deviation for the models obtained along the 100 runs performed using `cf-r` for the 109 stable and long-lived nuclides included in the training subset (left) and the remaining 145 stable nuclides of the nuclear chart with $A \geq 8$ included in the testing subset (right). It is possible to notice in both figures that lighter nuclides are more difficult to approximate. This fact can also be verified by deuterium and $^{1}_{2}$He, part of the remaining stable nuclides of the nuclear chart (testing subset). Their absolute average residual value was 2.354 MeV and 1.914 MeV, and the standard deviation was 11.834 MeV and 27.970 MeV, respectively. The heavier nuclides are better approximated by the overall `cf-r` models obtained in the 100 runs.

Descriptive statistics of the 100 independent runs with Dataset 1 and Dataset 2

For 109 stable isotopes - Dataset 1

Here we are investigating the effect of the value of Δ over Dataset 1. We performed 100 different independent runs to evaluate the results of either using $\Delta = 0$ and $\Delta = 0.1$. The results indicate that our approach is able to deliver reliable models. Moreover, the less complex model employing 3 features performed a bit better than the more complex model employing 5 features (see Supplementary Table S2).

To illustrate the performance difference between using $\Delta = 0$ and $\Delta = 0.1$ in the matter of MSE, Supplementary Fig. S3 shows the box plot comparing the results obtained using both approaches. It is possible to notice that using $\Delta = 0.1$ not only produced a smaller minimum MSE, but its variability is smaller too. According to the interquartile range, the median value of the results is smaller as well.

We also performed 10-fold cross-validation using the training subset of Dataset 1 to demonstrate the robustness of our method and to identify which nuclides are more difficult to model.

The nuclides were randomly split in each fold with 90% for training and 10% for testing. The results are presented in Supplementary Table S3. Observing the first roll it is possible to find the overall results, we can highlight the maximum testing

MSE	$\Delta = 0.1$	$\Delta = 0$
Max	3.687×10^{-2}	4.879×10^{-2}
Mean	1.326×10^{-2}	1.548×10^{-2}
Std Dev.	6.739×10^{-3}	9.507×10^{-3}
Min	2.805×10^{-3}	2.938×10^{-3}
Variables	**3**<br>$Z^{\frac{3}{2}}, N^{-1}, \delta(N,Z)$	5<br>$Z^{\frac{3}{2}}, Z^{-\frac{1}{2}}, A^{-\frac{1}{2}}, N^{-1}, \delta(N,Z)$

Supplementary Table S2. Statistical analysis of the results from 100 runs performed using a $depth_1$ CF aiming to evaluate two different values of the parameter Δ, used to penalise more complex models in the `cf-r`. The results show the efficiency of the method in selecting only the most meaningful features to obtain a simpler model and achieve a better approximation. Furthermore, it demonstrates that employing more variables in the model may not produce better approximations necessarily, we found a less complex model using 3 features that can outperform a more complex model using 5 features.

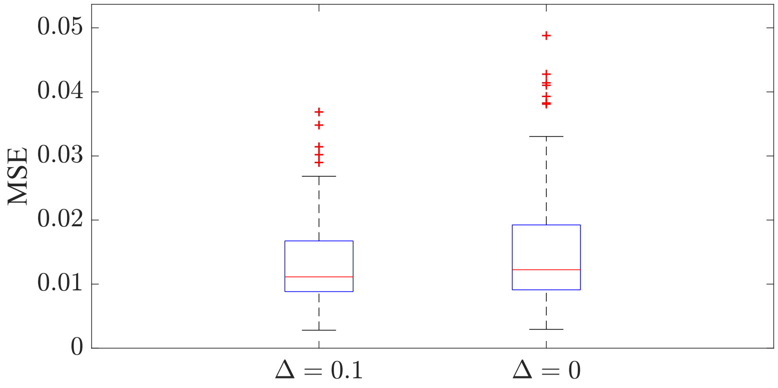

Supplementary Figure S3. Box plot illustrating the comparison of the performance over 100 runs using $\Delta = 0.1$ and $\Delta = 0$ on `cf-r` in the matter of minimizing the MSE for the reduced size group of stable and long-lived nuclides (training subset of Dataset 1). It is possible to notice that using $\Delta = 0.1$ not only produced a better minimum MSE, but its variability is smaller too, according to the interquartile range, and the median value of the results is smaller as well.

MSE is considerably high if compared to the minimum testing MSE obtained. This variability indicates that the model's performance is sensitive to the choice of nuclides in the training and testing subsets. After we investigated each fold result, we identified that Fold #1 is responsible for the worse performance in terms of MSE, due to the inclusion of the tritium ($^3_1$H) in the testing subset. The average absolute residual of the tritium on the 100 runs is 18.970 MeV, compared to the average absolute residual of 4.510×10^{-2} MeV from the remaining elements in the testing subset during the 100 runs. These results demonstrated how challenging is modeling the features of lighter nuclides like hydrogen and its isotopes.

	MSE	Min	Max	Mean	Std Dev.
Overall	Train	2.728×10^{-3}	1.266×10^{-1}	1.639×10^{-2}	1.190×10^{-2}
	Test	3.886×10^{-4}	8.017×10^{4}	8.302×10^{1}	2.535×10^{3}
Fold #1	Train	4.160×10^{-3}	3.317×10^{-2}	1.175×10^{-2}	5.097×10^{-3}
	Test	2.337×10^{-3}	8.017×10^{4}	8.290×10^{2}	8.015×10^{3}
Other folds	Train	2.728×10^{-3}	1.266×10^{-1}	1.691×10^{-2}	1.232×10^{-2}
	Test	3.886×10^{-4}	1.937	1.310×10^{-1}	3.151×10^{-1}

Supplementary Table S3. Statistical analysis in terms of MSE of the 10-fold cross-validation using a reduced size group of stable and long-lived nuclides of the nuclear chart present in Dataset 1. We present the overall result and specifically the results from the Fold #1 and the remaining 9 folds results, which performed 100 runs using a $depth_1$ CF. It is noticeable that the maximum overall MSE value is considerably higher than the minimum overall MSE value, contributing to an increase in the overall average MSE value. Investigating each fold result shows that Fold #1 is responsible for the worse performance in terms of MSE, due to the inclusion of the tritium ($^3_1$H) in the testing subset. The average absolute residual of the tritium on the 100 runs is 18.970 MeV, compared to the average absolute residual of 4.510×10^{-2} MeV from the remaining elements in the testing subset during the 100 runs. This demonstrates how challenging is modeling the features of lighter nuclides like hydrogen and its isotopes.

For Dataset 2

The initial investigation is over the use of sample weight to enhance the performance of our method on a specific region. The expression used to evaluate the weight used for each nuclide in the loss function ℓ_{Pen} is described in Section 'Results'. We employed a $depth_1$ CF for the training and testing subsets of Dataset 2.

Supplementary Table S4 shows the statistical analysis in terms of MSE of experiments exploring different scenarios, each performing 100 runs. Starting from left to right, in the first four columns we show the information from the experiments covering the whole dataset and not using sample weight to enhance the performance on lighter nuclides. Bringing more attention to the two columns in the center, they detail the results focusing on nuclides with $A \geq 200$. It seems that our approach is more effective for modeling heavier nuclides, as evidenced by the comparison between the statistical metrics listed in the two central columns and those in the first two columns, which encompass results for all nuclides. The final two columns present data from experiments that incorporated sample weighting. A slight enhancement is observable in the minimum value achieved with versus without the use of sample weighting. Nevertheless, the other statistical metrics suggest a decline in overall performance when sample weighting is applied.

MSE	No Sample Weight				Sample Weight	
	Dataset 2		Dataset 2 ($A \geq 200$)		Dataset 2	
	Train	Test	Train	Test	Train	Test
Min	3.190×10^{-2}	1.495×10^{-1}	1.541×10^{-3}	1.020×10^{-3}	3.121×10^{-2}	3.049×10^{-2}
Max	4.149×10^{-2}	1.765×10^{-1}	1.677×10^{-3}	3.124×10^{-3}	3.714×10^{-1}	8.738×10^{-1}
Mean	3.603×10^{-2}	1.598×10^{-1}	1.642×10^{-3}	2.545×10^{-3}	1.366×10^{-1}	3.081×10^{-1}
Std Dev.	4.229×10^{-3}	1.238×10^{-2}	6.724×10^{-5}	1.022×10^{-3}	7.969×10^{-2}	1.427×10^{-1}

Supplementary Table S4. Statistical analysis of the results in terms of MSE from 100 runs using `cf-r` and a $depth_1$ CF for the stable and unstable nuclides experimentally observed with $A \geq 8$ (training subset of Dataset 2) and unstable nuclides with estimated values (testing subset of Dataset 2) from the AME2020. In the first four columns from left to right, we show the information from the experiment covering the whole dataset and not using sample weight to enhance the performance on lighter nuclides. The two columns in the center detail the results focusing on nuclides with $A \geq 200$. Apparently, our method delivers better models for heavier nuclides, this is demonstrated when comparing the statistical indicators from the two columns in the center and the first two columns showing the results for all nuclides. The last two columns show the information from the experiment using sample weight. It is possible to verify a small improvement comparing the minimum value obtained using and not using sample weight. However, the remaining statistical indicators point to the fact that the overall performance deteriorates.

To illustrate the findings described in Supplementary Table S4 we used a box plot represented in Supplementary Fig. S4.

This plot illustrates that although using sample weighting reduced the minimum MSE obtained, the average performance was significantly worse. This fact is supported by observing the interquartile range of both approaches and also the distance of the worst result obtained.

Following the methodology described, we performed a 10-fold cross-validation using the experimentally observed nuclides (training subset) of Dataset 2 to demonstrate the robustness of our results obtained and to identify the presence of nuclides more difficult to model, as it was verified in Dataset 1.

The nuclides were randomly split in each fold with 90% for training and 10% for testing. According to the statistical analysis shown in Supplementary Table S4 that demonstrated a decrease in the performance when using sample weight, we have not used sample weight in the 10-fold cross-validation. The results are presented in Supplementary Table S5.

The statistical analysis shows consistency between the results reported in Supplementary Table S4 and the results reported in Supplementary Table S5. The fact that Dataset 2 includes nuclides with $A \geq 8$ and excludes lighter nuclides with different behavior benefits the modeling task. We can also verify that there are no specific experimentally observed nuclides deteriorating the performance of our method in Dataset 2, rather than reported in Supplementary Table S3 for the tritium in Dataset 1.

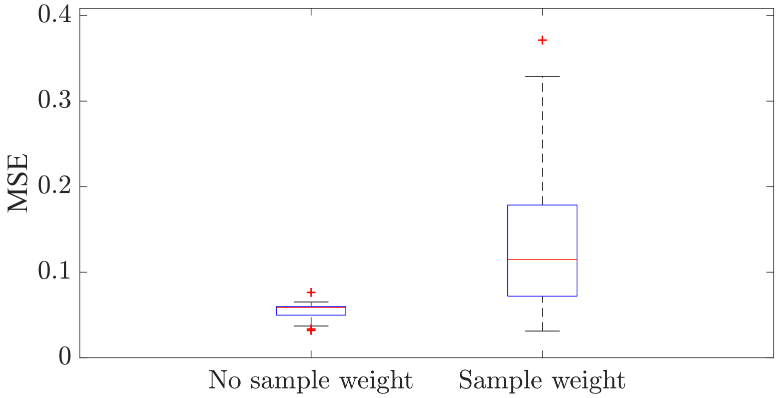

Supplementary Figure S4. Box plot illustrating the performance difference over the experimentally observed NBE values with $A \geq 8$ (training subset) and estimated values of NBE (testing subset). We analysed two distinct scenarios on `cf-r` in the matter of MSE, first not using weight on each sample (left) and second, using a weight for each sample and applying the loss function ℓ_{Pen} (right). This plot demonstrates that although using sample weighting produced the best model, the average performance was significantly worse. This fact is supported by observing the interquartile range of both approaches and also the distance of the worst result obtained.

	Subset	Min	Max	Mean	Std Dev.
MSE	Train	1.225×10^{-2}	8.860×10^{-2}	5.516×10^{-2}	8.990×10^{-3}
	Test	9.519×10^{-3}	8.413×10^{-1}	5.948×10^{-2}	3.537×10^{-2}

Supplementary Table S5. Statistical analysis in terms of MSE of the 10-fold cross-validation employing 90%/10% train/test rate of only the experimentally observed values of NBE of stable and unstable nuclides with $A \geq 8$ (training subset of Dataset 2). The statistical analysis shows consistency with the results reported in Supplementary Table S4. The fact that just nuclides with $A \geq 8$ are used, excluding lighter nuclides with different behavior, benefits the modeling task. We can also verify that there are no specific experimentally observed nuclides deteriorating the performance of our method in the group of experimentally observed values of NBE of stable and unstable nuclides with $A \geq 8$, rather than reported in Supplementary Table S3 for the stable and long-lived nuclides including those with $A < 8$.

Newton's Optimisation Method

We explore an approach to finding a solution for a problem based on the Newton-Raphson method. In the original method, we aim to find the root x of a function such that $f(x) = 0$. From this perspective, the optimisation method defines $g(x) = f'(x)$ according to the condition that the optimal value x^* satisfies either $g(x)$ and $f'(x)$ as $g(x^*) = f'(x^*) = 0$[38–40].

Considering a continuously differentiable function, the derivative can be evaluated and the optimisation problem can be expressed as a root-finding problem. In the case of a single variable function, Newton's method procedure to update the solution is defined as,

$$x_{k+1} = x_k - \frac{f'(x_k)}{f''(x_k)}, \tag{S6}$$

where k is the current iteration and, $f'(\cdot)$ and $f''(\cdot)$ are the first and second derivative, respectively. Supplementary Eq. (S6) can be adapted to a multi-variable function in the form of,

$$x_{k+1} = x_k - \nabla^2 f(x_k)^{-1}\,\nabla f(x_k), \tag{S7}$$

where $\nabla^2 f(x_k)$ is Hessian matrix and $\nabla f(x_k)$ is the Gradient matrix.

Description of the Nuclides included in Dataset 1

Nucleus	Z	A	N	B/A (EXP, MeV) 2020	Nucleus	Z	A	N	B/A (EXP, MeV) 2020
H[a]	1	3	2	2.827265	Ru	44	100	56	8.619359
He	2	4	2	7.073916	Ru	44	101	57	8.601366
Li	3	6	3	5.332331	Rh	45	103	58	8.584193
Li	3	7	4	5.60644	Pd	46	105	59	8.570651
Be	4	9	5	6.462669	Pd	46	106	60	8.579993
B	5	10	5	6.475084	Ag	47	107	60	8.553901
B	5	11	6	6.927732	Cd	48	110	62	8.551276
C	6	12	6	7.680145	Cd	48	111	63	8.53708
C	6	13	7	7.46985	In	49	113	64	8.52293
N	7	14	7	7.475615	Sn	50	115	65	8.51407
N	7	15	8	7.69946	Sn	50	116	66	8.523117
O	8	16	8	7.976207	Sb	51	121	70	8.482057
O	8	17	9	7.750729	Te	52	122	70	8.478132
F	9	19	10	7.779019	I	53	127	74	8.445482
Ne	10	21	11	7.971714	Xe	54	126	72	8.443538
Na	11	23	12	8.111494	Cs	55	133	78	8.409979
Mg	12	25	13	8.223503	Ba	56	132	76	8.409375
Mg	12	26	14	8.333871	Ba	56	134	78	8.408173
Al	13	27	14	8.331553	La[a]	57	138	81	8.375084
Si	14	29	15	8.448636	La	57	139	82	8.377999
Si	14	30	16	8.520655	Ce	58	138	80	8.377041
P	15	31	16	8.481168	Pr	59	141	82	8.353985
S	16	34	18	8.583499	Nd	60	143	83	8.330489
Cl	17	37	20	8.570282	Nd[a]	60	144	84	8.326924
Ar	18	38	20	8.614281	Sm	62	149	87	8.263468
K	19	41	22	8.576073	Sm	62	150	88	8.261624
Ca	20	43	23	8.600665	Eu	63	153	90	8.228701
Sc	21	45	24	8.618941	Gd	64	155	91	8.213254
Ti	22	47	25	8.661233	Gd	64	156	92	8.215325
Ti	22	48	26	8.723012	Tb	65	159	94	8.188803
V[a]	23	50	27	8.695903	Dy	66	160	94	8.184053
Cr	24	52	28	8.775995	Dy	66	161	95	8.173309
Mn	25	55	30	8.765025	Ho	67	165	98	8.146959
Fe	26	56	30	8.790356	Er	68	167	99	8.131735
Fe	26	57	31	8.770283	Tm	69	169	100	8.11447
Co	27	59	32	8.768038	Yb	70	173	103	8.087428
Ni	28	61	33	8.765028	Lu[a]	71	176	105	8.059021
Cu	29	63	34	8.75214	Hf	72	179	107	8.038547
Cu	29	65	36	8.757097	Hf	72	180	108	8.034932
Zn	30	66	36	8.759634	Ta	73	181	108	8.023405
Ga	31	69	38	8.72458	W	74	186	112	7.988603
Ge	32	70	38	8.721703	Re[a]	75	187	112	7.977952

Nucleus	Z	A	N	B/A (EXP, MeV) 2020	Nucleus	Z	A	N	B/A (EXP, MeV) 2020
As	33	75	42	8.700875	Os	76	192	116	7.948526
Se	34	76	42	8.711478	Ir	77	193	116	7.938135
Br	35	79	44	8.687596	Pt	78	198	120	7.914151
Kr	36	80	44	8.69293	Au	79	197	118	7.915655
Rb	37	85	48	8.697442	Hg	80	204	124	7.885546
Sr	38	84	46	8.677513	Tl	81	205	124	7.878395
Sr	38	86	48	8.708457	Pb	82	208	126	7.867453
Sr	38	88	50	8.732596	Bi[a]	83	209	126	7.847987
Y	39	89	50	8.714011	Th[a]	90	232	142	7.615034
Zr	40	90	50	8.70997	U[a]	92	234	142	7.600716
Nb	41	93	52	8.664185	U[a]	92	235	143	7.590915
Mo	42	94	52	8.662334	U[a]	92	238	146	7.570126
Mo	42	95	53	8.648721					

[a] Isotope not considered *stable*.

Supplementary Table S6. Details of the training subset of Dataset 1 selected from[22], showing the atomic number Z, atomic mass A, number of neutrons N, and the experimentally (EXP) observed value of the nuclear binding energy per nucleon B/A for the stable nuclides with the inclusion of tritium and other long-lived isotopes. (NuDat and AME2020[8])

Nuclide	Half-life	Nuclide	Half-life
$^{3}_{1}$H	12.32 y*	$^{187}_{75}$Re	4.33×10^{10} y
$^{50}_{23}$V	2.1×10^{17} y	$^{209}_{83}$Bi	2.01×10^{19} y
$^{132}_{56}$Ba	3.0×10^{21} y	$^{232}_{90}$Th	1.4×10^{10} y
$^{138}_{57}$La	1.02×10^{11} y	$^{234}_{92}$U	2.455×10^{5} y
$^{138}_{58}$Ce	$\geq 0.9 \times 10^{14}$ y	$^{235}_{92}$U	7.04×10^{8} y
$^{144}_{60}$Nd	2.29×10^{15} y	$^{238}_{92}$U	4.468×10^{9} y
$^{176}_{71}$Lu	3.76×10^{10} y		

* Measured value.

Supplementary Table S7. Detailed half-life of the nuclides not considered stable included in the training subset of Dataset 1 selected from[22]. All these nuclides are long-lived isotopes, with the exception of tritium (included due to its different physical properties). Half-lives obtained from NuDat and AME2020[8] are estimated, with the exception of tritium.

Description of the Stable Nuclides According to IAEA

Nucleus	Z	A	N	B/A (EXP, MeV) 2020	Nucleus	Z	A	N	B/A (EXP, MeV) 2020
H	1	2	1	1.112283	Pd	46	110	64	8.547163
He	2	3	1	2.572680	Ag	47	107	60	8.553901
He	2	4	2	7.073916	Ag	47	109	62	8.547916
Li	3	6	3	5.332331	Cd	48	106	58	8.539049
Li	3	7	4	5.606440	Cd	48	108	60	8.550020
Be	4	9	5	6.462669	Cd	48	110	62	8.551275
B	5	10	5	6.475084	Cd	48	111	63	8.537080
B	5	11	6	6.927732	Cd	48	112	64	8.544731
C	6	12	6	7.680145	Cd	48	114	66	8.531514
C	6	13	7	7.469849	In	49	113	64	8.522930
N	7	14	7	7.475615	Sn	50	112	62	8.513619
N	7	15	8	7.699460	Sn	50	114	64	8.522567
O	8	16	8	7.976207	Sn	50	115	65	8.514070
O	8	17	9	7.750729	Sn	50	116	66	8.523117
O	8	18	10	7.767098	Sn	50	117	67	8.509612
F	9	19	10	7.779019	Sn	50	118	68	8.516534
Ne	10	20	10	8.032241	Sn	50	119	69	8.499449
Ne	10	21	11	7.971714	Sn	50	120	70	8.504488
Ne	10	22	12	8.080466	Sn	50	122	72	8.487897
Na	11	23	12	8.111494	Sn	50	124	74	8.467400
Mg	12	24	12	8.260710	Sb	51	121	70	8.482057
Mg	12	25	13	8.223503	Sb	51	123	72	8.472320
Mg	12	26	14	8.333871	Te	52	120	68	8.476986
Al	13	27	14	8.331553	Te	52	122	70	8.478131
Si	14	28	14	8.447745	Te	52	124	72	8.473270
Si	14	29	15	8.448636	Te	52	125	73	8.458036
Si	14	30	16	8.520655	Te	52	126	74	8.463240
P	15	31	16	8.481168	I	53	127	74	8.445482
S	16	32	16	8.493130	Xe	54	126	72	8.443537
S	16	33	17	8.497630	Xe	54	128	74	8.443301
S	16	34	18	8.583499	Xe	54	129	75	8.431390
S	16	36	20	8.575390	Xe	54	130	76	8.437731
Cl	17	35	18	8.520279	Xe	54	131	77	8.423737
Cl	17	37	20	8.570282	Xe	54	132	78	8.427623
Ar	18	36	18	8.519910	Cs	55	133	78	8.409979
Ar	18	38	20	8.614281	Ba	56	130	74	8.405513
Ar	18	40	22	8.595259	Ba	56	134	78	8.408173
K	19	39	20	8.557026	Ba	56	135	79	8.397535
K	19	41	22	8.576073	Ba	56	136	80	8.402757
Ca	20	40	20	8.551305	Ba	56	137	81	8.391829
Ca	20	42	22	8.616565	Ba	56	138	82	8.393422
Ca	20	43	23	8.600665	La	57	139	82	8.377999

Nucleus	Z	A	N	B/A (EXP, MeV) 2020	Nucleus	Z	A	N	B/A (EXP, MeV) 2020
Ca	20	44	24	8.658177	Ce	58	136	78	8.373762
Ca	20	46	26	8.668985	Ce	58	140	82	8.376304
Sc	21	45	24	8.618941	Pr	59	141	82	8.353985
Ti	22	46	24	8.656462	Nd	60	142	82	8.346031
Ti	22	47	25	8.661233	Nd	60	143	83	8.330489
Ti	22	48	26	8.723012	Nd	60	145	85	8.309188
Ti	22	49	27	8.711163	Nd	60	146	86	8.304093
Ti	22	50	28	8.755723	Nd	60	148	88	8.277178
V	23	51	28	8.742085	Sm	62	144	82	8.303680
Cr	24	52	28	8.775995	Sm	62	149	87	8.263468
Cr	24	53	29	8.760210	Sm	62	150	88	8.261624
Cr	24	54	30	8.777967	Sm	62	152	90	8.244065
Mn	25	55	30	8.765025	Sm	62	154	92	8.226838
Fe	26	54	28	8.736385	Eu	63	153	90	8.228701
Fe	26	56	30	8.790356	Gd	64	154	90	8.224800
Fe	26	57	31	8.770283	Gd	64	155	91	8.213254
Fe	26	58	32	8.792253	Gd	64	156	92	8.215325
Co	27	59	32	8.768038	Gd	64	157	93	8.203507
Ni	28	58	30	8.732062	Gd	64	158	94	8.201823
Ni	28	60	32	8.780777	Gd	64	160	96	8.183017
Ni	28	61	33	8.765028	Tb	65	159	94	8.188802
Ni	28	62	34	8.794555	Dy	66	156	90	8.192437
Ni	28	64	36	8.777464	Dy	66	158	92	8.190130
Cu	29	63	34	8.752140	Dy	66	160	94	8.184053
Cu	29	65	36	8.757097	Dy	66	161	95	8.173309
Zn	30	64	34	8.735906	Dy	66	162	96	8.173455
Zn	30	66	36	8.759633	Dy	66	163	97	8.161784
Zn	30	67	37	8.734153	Dy	66	164	98	8.158713
Zn	30	68	38	8.755682	Ho	67	165	98	8.146959
Ga	31	69	38	8.724580	Er	68	162	94	8.152396
Ga	31	71	40	8.717605	Er	68	164	96	8.149019
Ge	32	70	38	8.721703	Er	68	166	98	8.141948
Ge	32	72	40	8.731746	Er	68	167	99	8.131735
Ge	32	73	41	8.705050	Er	68	168	100	8.129590
Ge	32	74	42	8.725201	Er	68	170	102	8.111953
Ge	32	76	44	8.705236	Tm	69	169	100	8.114470
As	33	75	42	8.700875	Yb	70	168	98	8.111887
Se	34	74	40	8.687715	Yb	70	170	100	8.106610
Se	34	76	42	8.711478	Yb	70	171	101	8.097883
Se	34	77	43	8.694691	Yb	70	172	102	8.097430
Se	34	78	44	8.717807	Yb	70	173	103	8.087428
Se	34	80	46	8.710814	Yb	70	174	104	8.083848
Br	35	79	44	8.687596	Yb	70	176	106	8.064085

Nucleus	Z	A	N	B/A (EXP, MeV) 2020	Nucleus	Z	A	N	B/A (EXP, MeV) 2020
Br	35	81	46	8.695946	Lu	71	175	104	8.069141
Kr	36	78	42	8.661238	Hf	72	176	104	8.061360
Kr	36	80	44	8.692930	Hf	72	177	105	8.051837
Kr	36	82	46	8.710675	Hf	72	178	106	8.049444
Kr	36	83	47	8.695730	Hf	72	179	107	8.038547
Kr	36	84	48	8.717447	Hf	72	180	108	8.034932
Kr	36	86	50	8.712029	Ta	73	181	108	8.023405
Rb	37	85	48	8.697442	W	74	182	108	8.018310
Sr	38	84	46	8.677513	W	74	184	110	8.005078
Sr	38	86	48	8.708457	W	74	186	112	7.988603
Sr	38	87	49	8.705236	Re	75	185	110	7.991010
Sr	38	88	50	8.732596	Os	76	187	111	7.973781
Y	39	89	50	8.714011	Os	76	188	112	7.973866
Zr	40	90	50	8.709970	Os	76	189	113	7.963003
Zr	40	91	51	8.693315	Os	76	190	114	7.962105
Zr	40	92	52	8.692678	Os	76	192	116	7.948526
Zr	40	94	54	8.666802	Ir	77	191	114	7.948114
Nb	41	93	52	8.664185	Ir	77	193	116	7.938135
Mo	42	92	50	8.657731	Pt	78	192	114	7.942492
Mo	42	94	52	8.662334	Pt	78	194	116	7.935942
Mo	42	95	53	8.648721	Pt	78	195	117	7.926553
Mo	42	96	54	8.653988	Pt	78	196	118	7.926530
Mo	42	97	55	8.635093	Pt	78	198	120	7.914151
Mo	42	98	56	8.635169	Au	79	197	118	7.915655
Ru	44	96	52	8.609413	Hg	80	196	116	7.914370
Ru	44	98	54	8.620314	Hg	80	198	118	7.911553
Ru	44	99	55	8.608713	Hg	80	199	119	7.905279
Ru	44	100	56	8.619359	Hg	80	200	120	7.905896
Ru	44	101	57	8.601366	Hg	80	201	121	7.897561
Ru	44	102	58	8.607428	Hg	80	202	122	7.896851
Ru	44	104	60	8.587400	Hg	80	204	124	7.885546
Rh	45	103	58	8.584193	Tl	81	203	122	7.886053
Pd	46	102	56	8.580289	Tl	81	205	124	7.878395
Pd	46	104	58	8.584848	Pb	82	206	124	7.875362
Pd	46	105	59	8.570651	Pb	82	207	125	7.869866
Pd	46	106	60	8.579993	Pb	82	208	126	7.867453
Pd	46	108	62	8.567024					

Supplementary Table S8. Details of the stable nuclides defined by IAEA. The table shows the atomic number Z, atomic mass A, number of neutrons N, and the experimentally (EXP) observed value of the nuclear binding energy per nucleon B/A. (AME2020[8] and NuDat).
